# Understanding Exercise Adherence: The Predictability of Past Experience and Motivational Determinants

**DOI:** 10.3390/brainsci10020098

**Published:** 2020-02-12

**Authors:** Filipe Rodrigues, Diogo S. Teixeira, Henrique P. Neiva, Luís Cid, Diogo Monteiro

**Affiliations:** 1 Sport Science School of Rio Maior–Polytechnique Institute of Santarém (ESDRM-IPSantarém), Rio Maior 2040-413, Portugal; frodrigues@esdrm.ipsantarem.pt (F.R.); luiscid@esdrm.ipsantarem.pt (L.C.); 2 Research Center in Sports, Health and Human Development (CIDESD), Vila Real 5001-801, Portugal; henriquepn@gmail.com; 3 Life Quality Research Center (CIEQV), Rio Maior 2040-413, Portugal; 4 University of Lusófona (ULHT), Lisbon 1749-024, Portugal; 5 Center for the Study of Human Performance (CIPER), Lisbon 1495-751, Portugal; 6 Department of Sports Science, Beira Interior University (UBI), Covilhã 6201-001, Portugal

**Keywords:** interpersonal behaviors, basic needs, behavioral regulation, past behavior, exercise adherence

## Abstract

Background: An unresolved debate lingers over the effect of past behavior on motivational patterns and future behavior stability in the exercise context. Theorists argue that past behavior has a residual effect on future behavior; however, empirical studies have shown that past behavior displays significant power in predicting behavior recurrence in the future. The present research aimed to examine the effect of past behavior and motivational determinants on future exercise adherence. Methods: Data from 437 Portuguese gym exercisers (female = 235; male = 202) aged between 18 and 53 years (M = 31.14; SD = 9.47), with exercise experience ranging from 6 to 12 months (M = 9.41; SD = 1.33) were considered for research. Participants completed a multi-section survey measuring interpersonal behaviors, basic psychological needs, behavioral regulations, and intentions. Data from past behavior and future exercise adherence were collected using computerized records of their attendance at the gym. Results: Positive and significant correlations paths were evidenced among perceived supportive behaviors, needs satisfaction, autonomous motivation, intentions and future exercise adherence. Similar results were presented among perceived thwarting behaviors, needs frustration, and controlled motivation. Regression paths showed that perceived supportive behavior, basic needs satisfaction, and autonomous motivation displayed positive and significant effects on future behaviors; thus, past behavior displayed the highest coefficient on future exercise adherence. Fitness professionals should aim at creating supportive environments, thus, improving the likelihood of being perceived by exercisers as need-supportive individuals. By doing so, as a result, exercisers would experience increased levels of autonomous motivation and higher rates of future exercise attendance at the gym. Hence, exercisers will gradually form their positive past exercise experience, increasing the probability of engaging in an exercise in the future.

## 1. Introduction

Physical inactivity leads to a pattern of chronic diseases in which individuals are at high risk of morbidity and early mortality [1,2]. According to recent surveys, over 46% of the European population is physically inactive, which represents an increase in a sedentary lifestyle when compared to previous surveys [3,4]. The number of people engaging in structured and consistent physical activity, such as exercising at the gym or in a health club, has been decreasing over the last years [5]. The Lack of motivation has been pointed out as one of the main reasons for physical inactivity, and so, most individuals of our society have never engaged in any health-related behaviors, such as exercise [3].

In the early stages, exercise participation requires effort and purpose to increase the chances for its repetition in the future [6]. Hence, repeating the behavior during a specific time period may lead to consistent exercise practice in the future [7]. While several empirical studies have shown that, when individuals exercise out of interest and self-determined motivation, they tend to maintain this behavior [8], little is known on whether past exercise frequency actually impacts future behavior [7]. This research proposes that the assessment of past behavior can assist in explaining exercise adherence by integrating its predictive power in a motivational sequence.

### 1.1. Theoretical Framework in the Exercise Context

Among various contemporary theories, the Self-Determination Theory (SDT) [9] stands out as one of the most studied frameworks in the exercise context [10]. This model considers motivational factors as agents for cognitive and behavioral outcomes, such as exercise commitment [11,12]. Central to SDT is the distinction between autonomous motivation, which refers to the experience of personal choice and autonomy, feeling that people’s actions represent their true self; and controlled motivation, which represents a feeling of being controlled, pressured or coerced into behaving according to external or self-imposed contingencies. SDT based reviews in the exercise context have shown that autonomous motivation has a positive and significant effect on intentions towards exercising in the future and continuous exercise adherence, whilst the controlled motivation seems to have a negative effect, representing an increase in drop-out rates [10,13].

The way individuals regulate their motivation towards exercising in the future is contingent upon the satisfaction or frustration of the three universal basic psychological needs (BPN) [14]. For this reason, autonomy (i.e., feelings of volitional choice and desire to be in control of one’s own life), competence (i.e., feelings of mastery and efficacy), and relatedness (i.e., feeling connected with others) must be satisfied to foster positive outcomes, such as autonomous motivation [15]. On the other hand, individuals can also experience BPN frustration, and therefore, increase the likelihood of negative consequences, such as ill-being [16] or controlled motivation and drop-out [17].

The level of BPN satisfaction and/or frustration is dependent on the extent of how individuals perceive active behaviors from peers and people in key positions that surround them (e.g., coaches, instructors, teachers). Consequently, perceived need-supportive behaviors (e.g., encouragement of personal choices, provision of positive feedback, and demonstration of emotional support) tend to promote BPN satisfaction in exercisers [10,18]. In contrast, perception of controlling/thwarting behaviors (e.g., making demands and imposing pressure, emphasizing guilt, and displaying cold and distant behaviors) leads to negative consequences, such as BPN frustration [19,20].

### 1.2. Intention and Past Behavior

Ajzen [21] posits that an individual’s intention towards a given behavior is the most proximal predictor of health-related outcomes [22]. However, results from previous research in the exercise context have shown that cognitive constructs tend to explain more variance in intention than in the behavior itself [23]. Thus, stronger intention does not directly predict increased behavior maintenance. In fact, individuals who have been exercising over a period of time could be less “dependent” on their intention and instead rely on their past experience to repeat the behavior it in the future, as shown by previous studies [24,25].

Although under-researched in the exercise context, past experience has been pointed out as a strong forecaster of future behavior [24]. This approach underpins the notion that higher levels of recapping the behavior in the past could increase the possibility for it to be repeated in the future, without high levels of motivation or purpose. In their meta-analysis, Hagger and Chatzisarantis [22] have shown that not only does past behavior predict future behavior, but it also acts as a motivational and cognitive determinant of behavior performance. Hence, it seems possible that past behavior could have a stronger effect on how often individuals will exercise in the future, considering their perceptions of interpersonal behaviors, BPN and motivation.

### 1.3. Past limitations and Agenda for Future Research

The predictive power of past behavior on future exercise adherence, considering both TPB and SDT frameworks, is scarce [26]. Since the majority of previous studies focused on other determinants of exercise [27], little is known about the impact of past behavior on motivational factors, intention and future behavior. This under-researched area needs to be developed in order to better understand how the mere occurrence of exercise can have an impact on exercisers repeating this behavior in the future. Furthermore, a specific and a practical question that needs to be addressed is whether perceived need-supportive behaviors are associated with future exercise practice, by having a sequential effect through basic needs, behavioral regulation, and intentions, and/or how a past repeated behavior can actually predict its recurrence in the future. Ergo, the aim of the present study was to analyze the predictive power of past behavior and motivational determinants on future exercise adherence.

## 2. Materials and Methods

Data from 437 Portuguese gym exercisers (female = 235; male = 202) aged between 18 and 53 years (M = 31.14; SD = 9.47), exercising in Portuguese gym and health clubs (*n* = 10) were considered for research. Participants had an exercise experience ranging from 6 to 12 months (M = 8.41; SD = 1.33) and the frequency of the training sessions ranged from two to five times per week (M = 2.36; SD = 0.74).

Ethical approval was obtained prior to data collection by the ethical committee (references number: CE-UBI-pJ-2018-044:ID683), and procedures performed in this study were in accordance with the 1964 Helsinki declaration and its later amendments or comparable ethical standards. Subsequently, several gym and health club managers (*n* = 10) were contacted, to whom the objectives of the study were explained. All of them authorized researchers to proceed with the data collection. Potential gym members were contacted at the front office and asked to voluntarily participate in the study. Researchers approached those who seemed to comply with the following inclusion criteria: Aged between 18 and 65 years; both genders; more or equal than six months and less or equal 12 months of exercise experience at that specific gym or health club. The rationale for the application of the 6 to 12 month criterion used in this study was based on existing literature: Individuals who have exercised regularly for at least six months present lower intentions to drop-out when compared to new gym members [28] Besides, drop-out rates are approximately 50% in the first six months, decreasing onwards from this period, according to Prochaska and DiClemente [6]. Those willing to participate signed an informed consent form, prior to filling out a multi-section survey. Informed consent was obtained from all individuals participating in this study. Gym managers did not interfere in the participants’ selection nor did they reward exercisers for their participation in this study. Participants filled the multi-section survey in approximately 20 min.

### 2.1. Measures

*Perceived Interpersonal Behaviors*. The Interpersonal Behavior Questionnaire in exercise (Portuguese version) [29] was used to assess supportive and thwarting behaviors perceived by exercisers regarding fitness instructors’ behaviors. This 24-item (six factors, four items each) instrument measures perceived need autonomy, competence, and related support, as well as perceived need autonomy, competence, and relatedness thwarting behaviors. Participants indicated their agreement with each statement using a 7-point scale ranging from 1 (*“not agree at all*”) to 7 (“*completely agree*”). Afterwards, composite scores were calculated for supportive and thwarting behaviors, as previously done in other studies [30,31].

*Basic Needs Satisfaction and Frustration*. The Basic Psychological Needs Satisfaction, and Frustration Scale in exercise (Portuguese version) [32] was used to measure BPN satisfaction and frustration exercisers experience during training. This 24-item (six factors, four items each) scale measures the experience of autonomy, competence, and relatedness satisfaction, as well as the experience of autonomy, competence, and relatedness frustration during exercise practice. Participants indicated their agreement with each statement item using a 5-point scale ranging from 1 (“*totally disagree*”) to 5 (“*totally agree*”). Afterwards, composite scores for BPN satisfaction and frustration were calculated according to previous studies [31,33].

*Behavioral Regulations*. The Behavioral Regulation Exercise Questionnaire (Portuguese version) [34] was used to assess how individuals regulate their behavior towards exercise. Specifically, these 18-items were used to measure amotivation, external regulation, introjected regulation, identified regulation, integrated regulation, and intrinsic motivation based on the SDT motivational continuum. Response options ranged from 0 (“*totally disagree*”) to 4 (“*totally agree*”), and composite scores for autonomous motivation (identified and integrated regulation, and intrinsic motivation) and controlled motivation (introjected and external regulation, and amotivation) were calculated based on previous assumptions [9,35].

Intention. We followed Ajzen’s [36] recommendation on creating a 3-item factor examining intention towards exercise adherence (e.g., “*I will continue to exercise frequently in the next six months as I currently do*”). Participants responded to each item using a 5-point Likert scale ranging from 1 (“*no, for sure*”) to 5 (“*yes, for sure*”).

*Exercise Adherence*. Computerized records at the gyms and health clubs were used to measure exercise adherence. Entries at the gym are monitored through turnstiles. Thus, for the present study, one entry at the gym accounted as one training session. As a consequence, the number of activities exercisers participated in during their visit at the gym was not of relevance and was counted as one training session. Past experience (gym entries six months before the initial assessment) and future exercise adherence (gym entries six months after the initial assessment) were considered for analysis. Similar procedures have already been used in the same context [11], when authors considered gym attendance records as an observable measure of behavior.

### 2.2. Statistical Analysis

Data were screened for outliers. Univariate (*z* > 3.00) and multivariate (D^2^ = *p*1 < 0.001, *p*2 < 0.001) outliers were excluded as recommended by Byrne [37]. Means (M), Standard Deviations (SD), Skewness (S), Kurtosis (K), and correlations were calculated for each variable. Composite Reliability (CR) coefficient was calculated using Raykov [38] formula and values ≥ 0.7 were considered as acceptable [39].

**Figure 1:**
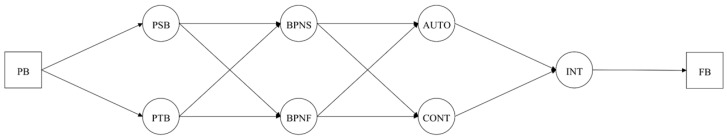
Hypothesized model with past behavior predicting perceived supportive and thwarting interpersonal behaviors. Note: PSB = Perceived Supportive Behaviors; PTB = Perceived Thwarting Behaviors; PB = Past Behavior; BPNS/F = Basic Psychological Needs Satisfaction/Frustration; AUTO = Autonomous Motivation; CONT = Controlled Motivation; INT = Intention; FB = Future Behavior.

**Figure 2:**
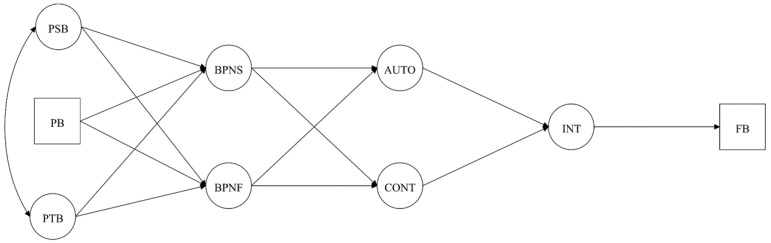
Hypothesized model with past behavior predicting basic psychological needs satisfaction and frustration. Note: PB = Past Behavior, PSB = Perceived Supportive Behaviors; PTB = Perceived Thwarting Behaviors; BPNS/F = Basic Psychological Needs Satisfaction/Frustration; AUTO = Autonomous Motivation; CONT = Controlled Motivation; INT = Intention; FB = Future Behavior.

**Figure 3:**
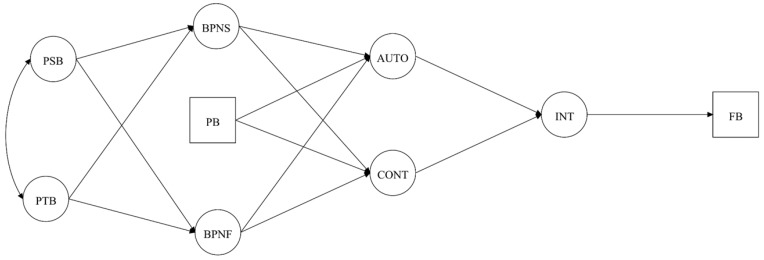
Hypothesized model with past behavior predicting autonomous and controlled motivation. Note: PSB = Perceived Supportive Behaviors; PTB = Perceived Thwarting Behaviors; PB = Past Behavior; BPNS/F = Basic Psychological Needs Satisfaction/Frustration; AUTO = Autonomous Motivation; CONT = Controlled Motivation; INT = Intention; FB = Future Behavior.

**Figure 4:**
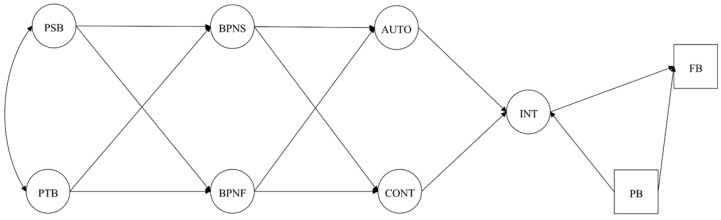
Hypothesized model with past behavior predicting intentions and future behavior. Note: PSB = Perceived Supportive Behaviors; PTB = Perceived Thwarting Behaviors; BPNS/F = Basic Psychological Needs Satisfaction/Frustration; AUTO = Autonomous Motivation; CONT = Controlled Motivation; INT = Intention; PB = Past Behavior; FB = Future Behavior.

Hypothesized models (Figure 1, Figure 2, Figure 3 and Figure 4) were analyzed using Mplus 7.4 [40] with the Robust Maximum Likelihood (MLR) estimator since it is robust to non-normality and non-independence of observations [41]. The chi-square statistics (*χ*^2^) and their respective degrees of freedom (*df*) were reported for visual orientation, but were not examined to assess model fit since these measures are sensitive to sample size and model specification [42]. For the CFA and SEM acceptance the following absolute and incremental indices were considered: Comparative Fit Index (CFI), Normalized Fit Index (NLI), Standard Root Mean Residual (SRMR), and Root Mean Square Error of Approximation (RMSEA) with its 90% Confidence Interval (CI 90%). For these indices, scores of CFI and TLI ≥ 0.90, SRMR and RMSEA ≤ 0.8 were considered acceptable [37,43,44]. Direct and indirect effects were analyzed according to standardized beta coefficients and its respective 95% Confidence Interval (CI 95%), accepting as significant if the confidence interval did not include zero [45].

## 3. Results

### 3.1. Preliminary Analysis

From the overall sample, 134 participants were excluded from further analysis since they did not meet some of the criteria: Aged above 65 years (*n* = 34); and had less than six months (*n* = 54) and more than 12 months (*n* = 46) of exercise experience. Several univariate (*n* = 1) and multivariate (*n* = 3) outliers were detected and excluded from further analysis. Descriptive statistics and the correlation matrix are presented in Table 1. Exercisers reported higher levels of perceived supportive behaviors, BPN satisfaction, and autonomous motivation compared to perceived thwarting behaviors, BPN frustration, and controlled motivation. Results displayed normal distribution since scores for skewness and kurtosis were contained within cutoffs. Perceived supportive behaviors, BPN satisfaction and autonomous motivation were positively and significantly correlated with each other, and negatively correlated with perceived thwarting behaviors, BPN frustration and controlled motivation. BPN satisfaction, autonomous motivation, and intention were positively correlated, whereas, BPN frustration and controlled motivation were negatively correlated with past behavior and future exercise adherence. Composite reliability coefficients were above cutoffs.

### 3.2. Main Analysis

Results from the goodness-of-fit indexes from all hypothetical models under analysis are shown in Table 2. Among the tested models, Model 4 (Figure 4), which considers past behavior as a predictor of intentions and future exercise adherence, was the only model presenting an acceptable fit. So, direct and indirect effects were assessed using model 4. As theoretically hypothesized, several significant effects were observed, namely: (i) Perceived supportive behaviors positively predicted BPN satisfaction; (ii) perceived thwarting behaviors positively predicted BPN frustration; (iii) BPN satisfaction positively predicted autonomous motivation; (iv) BPN frustration negatively predicted autonomous motivation and positively controlled motivation; (v) autonomous motivation was positively forecasted, whereas, controlled motivation negatively predicted intentions towards exercising; (vii) intentions and past behavior positively predicted future behavior. For detailed information, see Table 3.

Indirect effects of all possible regression paths among variables are displayed in Table 4. Perceived supportive behaviors showed a positive and significant effect on intentions and future behavior via BPN satisfaction and autonomous motivation. On the other hand, perceived thwarting behaviors negatively predicted intention towards exercising and future behavior through BPN frustration. BPN satisfaction displayed a positive and significant indirect effect on future behavior via autonomous motivation. However, BPN frustration showed a negative indirect effect on intentions and future behavior. Autonomous motivation and past behavior exhibited an indirect effect on future exercise adherence via intentions.

## 4. Discussion

The aim of the present study was to examine the effect of past behavior and motivational determinants on future exercise adherence. More specifically, the present study provides new insights on how motivational factors inherent to SDT interact with past behavior so as to predict future behavior. The obtained results may explain physical exercise maintenance in the future and will be discussed in light of previous research.

The present study tested four hypothesized models based on previous theoretical assumptions [22]. However, solely model 4 presented an acceptable fit. Regardless of perceived interpersonal behaviors, need satisfaction and frustration, and behavioral regulation, the observed effect of past behavior seems to only predict the intentions towards exercising and the performance of the behavior in the future. Consequently, past behavior does not play a moderation role in motivational determinants. Results suggest that individuals with previous exercise experience demonstrate a stable behavior on the long run, with past behavior acting as a strong predictor of future behavior.

Perceived need-supportive behaviors presented significant associations with BPN satisfaction and autonomous motivation. Additionally, perceived need-thwarting behaviors had a significant relationship with BPN frustration and controlled motivation. These results support theoretical [9] and empirical research in health-related behaviors [29,32,46], that preconizes the existence of a dual-process among motivational determinants. Autonomous motivation presented a positive and significant effect, whereas, controlled motivation displayed a negative and significant effect on intentions. These results are aligned with the ones from Ntoumanis et al. [12], suggesting that individuals who exercise, due to self-determined motivation and pleasure, and who consider exercising as an important behavior display stronger intentions to maintain this behavior in the future. Contrarily, it seems that individuals seeking external rewards or forcing themselves to exercise display weaker intentions towards exercising in the following 6-months after the initial assessment.

Intention positively and significantly predicted future exercise adherence; however, explained variance was lower when compared to the predictive power of past behavior. In fact, past exercise behavior presented the strongest prediction on future behavior when compared to intention. As a matter of fact, previous studies [47] posit that the effect of intention on behavior is likely to be affected by past behavior, when past behavior is included in the model. Thus, one might hypothesize that, in the absence of past behavior, intention directly influences behavior and motivation indirectly impacts behavior through intention, which might result in misleading conclusions regarding explained variance. Individuals who have been exercising over a period of time could be less “dependent” on their intention and instead rely on their past experience to repeat the behavior it in the future, as shown by previous studies [24,25]. As results show, it seems that past behavior could override the effect of motivational determinants on future exercise adherence. However, it is suggested that some intention is needed to promote future exercise adherence, as explained by the indirect effect of past behavior on future exercise adherence, via intention. Therefore, even though individuals seem to exercise because of their past experience, some levels of intentions towards exercising in the future could act as a mediator.

According to present results, consistently repeating the behavior could increase its frequency in the future [28]. Nevertheless, in order to obtain past exercise frequency, gym clients need to experience some levels of intentions to continue exercising, which is significantly predicted by the “bright” side of motivation (i.e., perceived need-supportive behaviors, BPN satisfaction, and autonomous motivation). Empirically, individuals engaging in exercise need to perceive fitness professionals as supportive (e.g., receive positive feedback from exercise instructors and feel connected with them), in order to experience higher levels of BPN satisfaction, and autonomous motivation, respectively. Ryan and Deci [9] have theoretically proven this, and several studies on sports [48], physical education [20], and exercise [49] are in conformity with present results.

Results exhibited significant negative indirect effects of the “dark” side of motivation (perceived thwarting behaviors, BPN frustration, and controlled motivation), on future exercise behavior. Imbalanced behavior performance, such as “*I feel pressured by my exercise instructor so I will exercise today*” and “*I will not exercise tomorrow because by exercise instructor is always complaining about my technique and he does not feel empathy*” might lead to a decrease in intention towards exercising, eventually resulting in withdrawal episodes or even drop-outs. Past studies have shown that supervisors (e.g., teachers, coaches) who use need-thwarting or need-controlling behaviors were perceived as accountable for BPN frustration [19,50]. Thus, controlled forms of motivation and BPN frustration is likely to be related to external or self-induced forces affecting exercisers behavior, lowering their intention to maintain the behavior in the future.

Lally and colleagues [51] proposed that repeating a behavior, for instance, exercising, in response to specific cues (e.g., feeling supported by exercise instructors) could foster its performance in the future. However, the required level of consistency and the time frame for it to become “habitual” has not been fully scrutinized. Studies regarding behavior stability [6,28] propose that the ratio of drop-out decreases by approximately 50% after six months. The present sample of participants had more than six months of the exercise experience. On that account, it is possible that their intentions towards exercising could be sustained with past exercise adherence. In fact, past behavior explained 85% of future behavior, a ratio above the one found by previous studies [52]. Differences between the present and past results may be related to implications from other processes involved. One possible explanation is that data from past exercise frequency from individuals exercising at gyms and health clubs are likely to be more easily gathered than data from other behaviors, such as healthy eating, blood donation or tooth brushing. Since exercising at a gym needs explicit preparation cues (e.g., preparing the gym bag, drive or walk to the gym) and performance (e.g., working out, following training plan), their repetition could be based on how previous experience was. Contrarily, other behaviors, such as drinking a glass of water could be less dependent on past experience and motivational factors, since they do not require any preparation cues and are facilitated by the easiness of the behavior [51]. Nevertheless, past behavior and frequency (e.g., going to the gym three times a week) are not sufficient to determine whether the behavior is a routine or not. Moreover, the conditions in which or due to which the behavior is performed might also broaden our understanding of why individuals exercise on the long run (e.g., what is my objective by going to the gym? And why am I still doing this?). Therefore, understanding cues like motivation are paramount to understand why individuals engage in regular exercise. The intention may not be sufficient to develop an unconscious process of behavior repetition in the future.

Among the strengths of the present research is the use of objective measurement of exercise behavior and the follow-up design. Results confirm the predictive value of past behavior on future exercise behavior [24], both directly and indirectly via intention. In addition, we support Hagger’s [7] prospect for future research by showing that motivational antecedents predict intention future exercise adherence.

### Limitations and Agenda for Future Research

First of all, this study was conducted within the exercise context considering data from a Portuguese sample of experienced exercisers. Thus, we cannot generalize these results to other domains. Additionally, potential participants who refused to participate were not counted. Their participation in this study could have presented different results compared to the current ones presented. Another limitation of the present study is that the cross-sectional approach with follow-up is limited to explaining exercise maintenance based on past behavior and motivational determinants. Future randomized controlled trial research is needed to sustain these results and consequently develop effective interventions. Secondly, this study used composite scores of the motivational determinants to avoid collinearity problems, so the results are limited to the compound scores from each construct. Moreover, demographic parameters, such as culture and age range could have influenced current results, since motivation could vary among exercisers with different characteristics [47]. Thus, forthcoming studies should fill this gap by testing this model on larger samples, as well as on the samples with different cultural background. Although current results point to a possible role of exercise frequency as a moderator of intention and future behavior, testing the effects of other key aspects, such as habit formation and behavior automaticity, past-future behavior prediction and intention-behavior relations are recommended for future research.

## 5. Conclusions

Notwithstanding the fact that initiating an exercise program is crucial for a healthy lifestyle, creating interventions that help society members to adhere to long-term physical exercise participation are paramount. Current results suggest that past behavior, directly and indirectly, predict, through intention, the future behavior to exercise over the subsequent six months. The present study evidences the relevant role of need-supportive behaviors in leading individuals to regularly repeat the behavior and provides health promoters and researchers with some guidelines on how to encourage individuals to be physically active in the future. Therefore, regularly repeating the behavior with perceived support from exercise instructors could create favorable conditions to promote long-term exercise adherence.

## Figures and Tables

**Table 1:** Descriptive statistics, composite reliability, and correlation matrix.

	M	SD	S	K	CR	Correlation Matrix								
						1	2	3	4	5	6	7	8	9
1. Perceived Supportive Behaviors	5.02	0.79	−0.18	0.04	0.80	1								
2. Perceived Thwarting Behaviors	2.49	0.87	0.28	−0.11	0.72	−0.66 **	1							
3. BPN Satisfaction	4.04	0.51	−0.58	1.6	0.75	0.63 **	−0.25 **	1						
4. BPN Frustration	1.80	0.57	0.74	0.41	0.76	−0.46 **	0.58 **	−0.81 **	1					
5. Autonomous Motivation	3.32	0.49	−0.57	0.46	0.77	0.52 **	−0.35 **	0.56 **	−0.54 **	1				
6. Controlled Motivation	0.04	0.34	0.67	−0.17	0.70	−0.31 **	0.57 **	−0.30 **	0.61 **	−0.61 **	1			
7. Intention	4.46	0.71	−1.58	2.21	0.94	0.27 **	−0.23 **	0.37 **	−0.30 **	0.45 **	−0.35 **	1		
8. Past Behavior	69.52	29.74	0.67	−0.02	-	0.10 **	0.07	0.11 *	−0.14 *	0.28 **	−0.17 **	0.21 **	1	
9. Future Exercise Adherence	67.59	31.58	0.47	−0.29	-	0.11 **	0.05	0.10 *	−0.10 *	0.27 **	−0.16 **	0.24 **	0.86 **	1

M = Mean; SD = Standard Deviation; S = Skewness; K = Kurtosis; CR = Composite Reliability; * *p* < 0.05; ** *p* < 0.01.

**Table 2:** Model fit indexes.

	*χ* ^2^	*df*	CFI	NFI	SRMR	RMSEA	90% CI
Figure 1	1209.663 *	198	0.63	0.57	0.13	0.13	0.12, 0.14
Figure 2	1100.716 *	197	0.67	0.61	0.09	0.12	0.11, 0.13
Figure 3	1091.767 *	197	0.67	0.62	0.09	0.12	0.11, 0.13
Figure 4	456.379 *	197	0.91	0.89	0.07	0.07	0.06, 0.08

CFI = Comparative Fit Index; NFI = Normalized Fit Index (NNFI); SRMR = Standard Root Mean Residual; RMSEA = Root Mean Square Error of Approximation (RMSEA); * significant at *p* < 0.01.

**Table 3:** Regression paths among constructs.

	β	SE	CI95%	
			Lower	Upper
Supportive Behaviors → BPN Satisfaction	0.72	0.20	0.38	0.89
Supportive Behaviors → BPN Frustration	−0.31	0.19	−0.63	0.02
Thwarting Behaviors → BPN Satisfaction	0.14	0.21	−0.21	0.49
Thwarting Behaviors → BPN Frustration	0.41	0.20	0.08	0.74
BPN Satisfaction → Autonomous Motivation	0.36	0.12	0.16	0.56
BPN Satisfaction → Controlled Motivation	0.07	0.16	−0.20	0.34
BPN Frustration → Autonomous Motivation	−0.43	0.09	−0.59	−0.27
BPN Frustration → Controlled Motivation	0.73	0.13	0.52	0.94
Autonomous Motivation → Intention	0.36	0.08	16	0.56
Controlled Motivation → Intention	−0.14	0.11	−0.34	−0.05
Intention → Future Behavior	0.04	0.02	0.04	0.08
Past Behavior → Intention	0.12	0.05	0.03	0.20
Past Behavior → Future Exercise Adherence	0.92	0.01	0.89	0.95

β = standardized coefficient; SE = standard error; CI95% = Confidence Interval at 95%.

**Table 4:** Indirect paths among constructs.

	β	SE	CI95%	
			Lower	Upper
Supportive Behaviors → BPNS → AUTO → INT → FEA	0.09	0.01	0.04	0.18
Supportive Behaviors → BPNS → CONT → INT → FEA	0.01	0.01	−0.01	0.03
Supportive Behaviors → BPNF → AUTO → INT → FEA	−0.01	0.00	−0.01	0.03
Supportive Behaviors → BPNF → CONT → INT → FEA	0.01	0.01	−0.01	0.02
Thwarting Behaviors → BPNS → AUTO → INT → FEA	0.01	0.01	−0.02	0.03
Thwarting Behaviors → BPNS → CONT → INT → FEA	−0.02	0.01	−0.13	0.09
Thwarting Behaviors → BPNF → AUTO → INT → FEA	−0.11	0.00	−0.31	−0.09
Thwarting Behaviors → BPNF → CONT → INT → FEA	−0.08	0.02	−0.20	−0.02
Supportive Behaviors → BPNS → AUTO → INT	0.10	0.05	0.02	0.17
Supportive Behaviors → BPNS → CONT → INT	0.05	0.04	−0.01	0.11
Supportive Behaviors → BPNF → AUTO → INT	−0.01	0.02	−0.04	0.02
Supportive Behaviors → BPNF → CONT → INT	0.03	0.03	−0.02	0.09
Thwarting Behaviors → BPNS → AUTO → INT	0.02	0.03	−0.03	0.07
Thwarting Behaviors → BPNS → CONT → INT	−0.07	0.04	−0.13	−0.01
Thwarting Behaviors → BPNF → AUTO → INT	0.00	0.00	−0.01	0.01
Thwarting Behaviors → BPNF → CONT → INT	−0.09	0.04	−0.10	−0.02
Supportive Behaviors → BPNS → AUTO	0.25	0.11	0.08	0.43
Supportive Behaviors → BPNS → CONT	0.05	0.07	−0.15	0.25
Supportive Behaviors → BPNF → AUTO	0.13	0.09	0.02	0.29
Supportive Behaviors → BPNF → CONT	−0.22	0.08	−0.48	−0.09
Thwarting Behaviors → BPNS → AUTO	0.05	0.12	−0.07	0.17
Thwarting Behaviors → BPNS → CONT	0.01	0.03	−0.04	0.07
Thwarting Behaviors → BPNF → AUTO	−0.18	0.15	−0.32	−0.03
Thwarting Behaviors → BPNF → CONT	0.30	0.16	0.04	0.56
BPNS → AUTO → INT → FEA	0.11	0.02	0.02	0.21
BPNS → CONT → INT → FEA	−0.02	0.01	−0.01	0.01
BPNF → AUTO → INT → FEA	−0.04	0.02	−0.07	−0.01
BPNF → CONT → INT → FEA	−0.03	0.02	−0.06	−0.01
BPNS → AUTO → INT	0.14	0.05	0.05	0.23
BPNS → CONT → INT	−0.01	0.02	−0.05	0.03
BPNF → AUTO → INT	−0.11	0.05	−0.23	−0.01
BPNF → CONT → INT	−0.16	0.08	−0.25	−0.08
AUTO → INT → FEA	0.09	0.03	0.04	0.14
CONT → INT → FEA	−0.04	0.03	−0.08	0.01
PB → INT → FEA	0.10	0.01	0.02	0.19

β = standardized coefficient; SE = standard error; CI95% = Confidence Interval at 95%.
